# Safety, tolerability, pharmacokinetics, and pharmacodynamics of the afucosylated, humanized anti-EPHA2 antibody DS-8895a: a first-in-human phase I dose escalation and dose expansion study in patients with advanced solid tumors

**DOI:** 10.1186/s40425-019-0679-9

**Published:** 2019-08-14

**Authors:** Kohei Shitara, Taroh Satoh, Satoru Iwasa, Kensei Yamaguchi, Kei Muro, Yoshito Komatsu, Tomohiro Nishina, Taito Esaki, Jun Hasegawa, Yasuyuki Kakurai, Emi Kamiyama, Tomoko Nakata, Kota Nakamura, Hayato Sakaki, Ichinosuke Hyodo

**Affiliations:** 1 National Cancer Center Hospital East, 6-5-1 Kashiwanoha, Kashiwa City, Chiba Japan; 2 Osaka University Graduate School of Medicine, Osaka, Japan; 3 National Cancer Center Hospital, Tokyo, Japan; 4 Cancer Institute Hospital of Japan Foundation for Cancer Research, Tokyo, Japan; 5 Aichi Cancer Center Hospital and Research Institute, Aichi, Japan; 6 Hokkaido University Hospital, Hokkaido, Japan; 7 National Shikoku Cancer Center Hospital, Ehime, Japan; 8 National Hospital Organization Kyushu Cancer Center, Fukuoka, Japan; 9 Daiichi Sankyo Co., Ltd, Tokyo, Japan; 10 University of Tsukuba, Ibaraki, Japan

**Keywords:** Erythropoietin-producing hepatocellular receptor A2, Gastric cancer, Esophageal cancer, Advanced solid tumors, DS-8895a, Antibody-dependent cellular cytotoxicity, Phase I study

## Abstract

**Background:**

Erythropoietin-producing hepatocellular receptor A2 (EPHA2) is overexpressed on the cell surface in many cancers and predicts poor prognosis. DS-8895a is a humanized anti-EPHA2 IgG1 monoclonal antibody afucosylated to enhance antibody-dependent cellular cytotoxicity activity. We conducted a two-step, phase I, multicenter, open-label study to determine the safety, tolerability, and pharmacokinetics of DS-8895a in patients with advanced solid tumors.

**Methods:**

Step 1 was a dose escalation cohort in advanced solid tumor patients (six dose levels, 0.1–20 mg/kg) to determine Step 2 dosing. Step 2 was a dose expansion cohort in EPHA2-positive esophageal and gastric cancer patients. DS-8895a was intravenously administered every 2 weeks for the duration of the study, with a 28-day period to assess dose-limiting toxicity (DLT). Safety, pharmacokinetics, tumor response, and potential biomarkers were evaluated.

**Results:**

Thirty-seven patients (Step 1: 22, Step 2: 15 [9: gastric cancer, 6: esophageal cancer]) were enrolled. Although one DLT (Grade 4 platelet count decreased) was observed in Step 1 (dose level 6, 20 mg/kg), the maximum tolerated dose was not reached; the highest dose (20 mg/kg) was used in Step 2. Of the 37 patients, 24 (64.9%) experienced drug-related adverse events (AEs) including three (8.1%) with Grade ≥ 3 AEs. Infusion-related reactions occurred in 19 patients (51.4%) but were manageable. All patients discontinued the study (evident disease progression, 33; AEs, 4). Maximum and trough serum DS-8895a concentrations increased dose-dependently. One gastric cancer patient achieved partial response and 13 patients achieved stable disease. Serum inflammatory cytokines transiently increased at completion of and 4 h after the start of DS-8895a administration. The proportion of CD16-positive natural killer (NK) cells (CD3^−^CD56^+^CD16^+^) decreased 4 h after the start of DS-8895a administration, and the ratio of CD3^−^CD56^+^CD137^+^ to CD3^−^CD56^+^CD16^+^ cells increased on day 3.

**Conclusions:**

Twenty mg/kg DS-8895a infused intravenously every 2 weeks was generally safe and well tolerated in patients (*n* = 21) with advanced solid tumors. The exposure of DS-8895a seemed to increase dose-dependently and induce activated NK cells.

**Trial registration:**

Phase 1 Study of DS-8895a in patients with advanced solid tumors (NCT02004717; 7 November 2013 to 2 February 2017); retrospectively registered on 9 December 2013.

**Electronic supplementary material:**

The online version of this article (10.1186/s40425-019-0679-9) contains supplementary material, which is available to authorized users.

## Background

Erythropoietin-producing hepatocellular receptor A2 (EPHA2) is a 130 kDa type-I transmembrane tyrosine kinase receptor, and EPH-related receptor tyrosine kinase ligand A1 (EPHRIN-A1) is the principle ligand for EPHA2 [1]. EPHA2/EPHRIN-A1 signaling contributes to the maintenance of epithelial cell homeostasis [1–3]. EPHA2 is expressed in several normal human tissues including skin, colon, bladder, kidney, lung, and stomach [4–6]. EPHA2 is overexpressed in many types of cancers [7–13], including 60% of gastric cancers [5] and nearly 50% of esophageal cancers [14]. It is widely reported that EPHA2 overexpression is correlated with poor prognosis for cancer patients [1, 4, 15–17]. Overexpressed EPHA2 promotes tumor cell proliferation, migration, invasion, and metastasis; and EPHA2 is activated through phosphorylation at serine-897 by AKT, p90 ribosomal S6 kinases, and protein kinase A, but not by EPHRIN-A1 [1–3, 18, 19]. In addition, Ras-Erk signaling, which is frequently activated in aggressive tumors, promotes expression of EPHA2 [20]. These features of EPHA2 make it an attractive target for cancer therapy.

Fragment crystallizable gamma receptor IIIa (FcγRIIIa, CD16) is highly expressed on natural killer (NK) cells. FcγRIIIa/CD16 binds to the Fc portion of IgG antibodies and induces the release of perforin and granzyme upon antibody/target cell binding, resulting in the death of target cells. This process is called antibody-dependent cellular cytotoxicity (ADCC). Afucosylation of the carbohydrate chain in IgG1 Fc substantially potentiates the binding affinity of the IgG1 portion to FcγRIIIa/CD16, which results in enhancement of ADCC [21, 22]. DS-8895a is a humanized anti-EPHA2 IgG1 monoclonal antibody (Additional file 1) that is afucosylated to enhance ADCC (POTELLIGENT®; BioWa Inc., Princeton, NJ, USA) and is expected to produce antitumor effects on EPHA2-overexpressing tumor cells through ADCC, as demonstrated in pre-clinical studies [23]. DS-8895a had neither complement-dependent cytotoxicity nor agonist activity against EPHA2 in vitro*,* and only weakly inhibited EPHRIN-A1-mediated phosphorylation of EPHA2 [23]. ADCC function is associated with antigen density [24], and overexpression of EPHA2 in solid tumors is considered a suitable and promising target for the ADCC-enhanced antibody DS-8895a.

The promising findings in pre-clinical studies led us to the clinical development of DS-8895a. We aimed to assess the safety, tolerability, and pharmacokinetics (PK) of DS-8895a administered in repeat doses to patients with advanced solid tumors and EPHA2-positive gastric or esophageal cancer in this first-in-human study of DS-8895a. Additionally, tumor response and potential biomarkers of tumor response were explored.

## Methods

### Study objectives

The primary objectives of this phase I, multicenter, open-label study, were to assess the safety, tolerability, and PK of repeated dosing of DS-8895a in patients with advanced solid tumours and to determine its optimal dose for subsequent clinical studies. The secondary objectives were to explore tumor response to DS-8895a treatment and the potential biomarkers related to DS-8895a.

### Patients

Inclusion criteria were as follows: advanced solid tumors in Step 1, immunohistologically confirmed EPHA2-positive gastric or esophageal cancer in Step 2, refractory to standard treatment or no standard treatment available, age ≥ 20 years old, Eastern Cooperative Oncology Group performance status ≤1, sufficient organ function within 7 days prior to registration (Additional file 2), adverse drug reaction of prior anti-cancer therapy resolved to Grade 1 or Grade 2 and assessed as clinically eligible by investigators, certain treatment-free period from the final dose/treatment of any previous therapy to the date of registration (Additional file 3), life expectancy ≥3 months, and written informed consent to the study including agreement to biomarker analysis of archival and biopsied tumor samples. A tumor was considered EPHA2-positive if ≥25% of tumor cells had weak to moderate (score 2+) or strong (3+) EPHA2 staining immunohistochemically.

Major exclusion criteria were as follows: symptomatic or treatment-required brain metastasis within 6 months of registration; positive for hepatitis B surface antigen, hepatitis C virus, or human immunodeficiency virus antibody; active gastrointestinal hemorrhage requiring blood transfusions within 2 weeks of registration; treatment with other investigational drugs within 3 weeks of registration; lactating or pregnant mothers; and unwillingness to use adequate contraception during the study and for 6 months after the final DS-8895a administration.

### Study design and treatment

The study protocol, amendments, and informed consent forms were approved by the Institutional Review Boards at each study site, and the study was conducted in accordance with the ethical principles of the Declaration of Helsinki and the International Council for Harmonisation guideline for Good Clinical Practice, and followed all other applicable regulatory requirements in Japan. Research using samples for genome/gene analysis was conducted in compliance with the Ethical Guidelines for Human Genome/Gene Analysis Research [25] and the Ethical Guideline for Clinical Research [26] as well as the above guidelines. This study was registered at clincaltrials.gov (NCT02004717).

This phase I study conducted in Japan comprised two steps: Step 1 as the dose escalation cohort in patients with advanced solid tumors, and Step 2 as the dose expansion cohort in patients with EPHA2-positive esophageal and gastric cancers (Additional file 4). In Step 1, the dose of DS-8895a was sequentially increased from Level 1 (0.1 mg/kg) to Level 6 (20 mg/kg), and intravenously administered over 2 h every 2 weeks with a 28-day period for assessment of dose-limiting toxicity (DLT). Each dose level consisted of three or six patients. If no DLT was observed in the first three patients, the dose level was escalated. If a DLT occurred in 1/3 patients, three patients were added to that dose level. If 2/6 or 3/6 patients experienced a DLT, dose escalation was discontinued and the dose level was judged as maximum tolerated dose (MTD). If the first 2/3 or 3/3 patients experienced a DLT, this dose level was also judged as MTD. In this case, further evaluation of the previous dose level was conducted, adding three or more patients. If the initial dose level was MTD, the study was stopped. Intra-patient dose escalation was not allowed. DLTs are defined in Additional file 5. Infusion-related reactions (IRRs) were excluded from the DLT evaluation.

The starting dose and administration schedule of DS-8895a were determined based on data from unpublished studies of intravenous administration of DS-8895a to cynomolgus monkeys (data on file, Daiichi Sankyo Co., Ltd.). Starting dose of 0.1 mg/kg was 260 times lower than the calculated human equivalent dose of no-observed-adverse-effect level obtained in these studies. An administration schedule of 2 weeks was chosen based on PK in cynomolgus monkeys and mice after a single intravenous or intraperitoneal administration of DS-8895a, respectively.

In Step 2, safety and PK were evaluated in up to 20 patients at the dose determined in Step 1. In both Steps 1 and 2, one cycle consisted of 4 weeks, and multiple cycles were allowed unless the discontinuation criteria for an individual patient were met.

Discontinuation criteria included evident disease progression, an adverse event (AE) making treatment continuation difficult, postponement of the study treatment > 4 weeks, deviation from the inclusion criteria after registration, patient’s request of withdrawal from study treatment, and investigator’s judgement.

Administration of DS-8895a was postponed if the patients did not meet the following criteria: ≥1000/μL neutrophil count, ≥75000/μL platelet count, non-hematological toxicity ≤ Grade 2 or improvement to the baseline level. Cancer treatment other than DS-8895a was prohibited from the day of obtaining informed consent to the day of follow-up assessment (30–45 days after final administration). Treatment for concomitant symptoms of cancer was allowed.

### Safety and tolerability assessments

All AEs, clinical laboratory tests (hematology, blood chemistry, and urinalysis), vital signs (systolic and diastolic blood pressure, pulse rate, body temperature), and electrocardiogram (ECG) were assessed according to the National Cancer Institute – Common Terminology Criteria for Adverse Events, version 4.0 (Japanese version).

Initially, no premedication was used to prevent IRRs. However, after IRRs were observed at dose Levels 1 and 2, the protocol was amended to allow a premedication with antihistamines and antipyretics in Level 3, and a premedication with additional corticosteroids in Level 4 and thereafter. If no IRR occurred in previous DS-8895a administration, it could be omitted from subsequent doses.

### Pharmacokinetic assessments

In Cycles 1 and 2, blood samples for PK analysis were collected immediately before and after DS-8895a administration; 4, 7, 24, and 72 h after starting administration on day 1; any time point on day 8; immediately before and after the next DS-8895a administration on day 15; and immediately before subsequent DS-8895a administration on day 29. From Cycle 3, blood samples were taken immediately before and after DS-8895a administration on day 1. Blood was also collected on the day of study discontinuation and on the 30th day after the final dose.

The serum concentrations of DS-8895a in Cycles 1 and 2 were used to calculate PK parameters (maximum serum concentration [C_max_], time to reach maximum serum concentration [T_max_], area under the concentration-time curve [AUC_last_] up to the last quantifiable time, AUC during dosing interval, AUC up to infinity [AUC_inf_], and terminal elimination half-life [T_1/2_]) using non-compartment models and the WinNonlin® software program (Certara, Princeton, USA). The lower limit of detection was set at 1000 ng/mL.

### Pharmacodynamics assessments

Archival tumor samples were assessed for EPHA2, E-cadherin, human epidermal growth factor receptor 2 (HER2), and epidermal growth factor receptor (EGFR) expression (Steps 1 and 2). In Step 2, tumor biopsy samples were obtained before Cycle 1 and 2, and EPHA2, CD16, NKp46/NCRI, CD3, CD68, programmed death ligand-1 (PD-L1), E-cadherin, EGFR, and HER2 expression were assessed by immunohistochemistry. EPHA2 was detected using anti-human EPHA2 mouse monoclonal antibodies (clones 018 and 058, Daiichi Sankyo Co., Ltd.).

Blood and serum samples were collected to test circulating CD16-positive NK cells, NK activity, human leukocyte antigen (HLA)/killer cell immunoglobin-like receptor (KIR) mismatch, cytokines, and soluble EPHA2; a detailed blood sampling schedule is given in Additional file 6. *HLA/KIR* mismatch was assessed by typing these genes using previously described methods [27]. Circulating CD16-positive NK cells (CD3^−^CD56^+^CD16^+^, in Steps 1 and 2) and the ratio of CD3^−^CD56^+^ CD137^+^ cells to CD3^−^CD56^+^CD16^+^ cells (only in Step 2) in blood samples were analyzed using flow cytometry. Circulating soluble EPHA2 (only in Step 2) in serum was analyzed by sandwich ELISA. Cytokines (granulocyte-macrophage colony-stimulating factor, interferon [IFN]γ, interleukin [IL]-2, IL-3, IL-4, IL-5, IL-6, IL-7, IL-8, IL-10, IL-18, macrophage inflammatory protein [MIP]-1α, MIP-1β, monocyte chemotactic protein [MCP]-1, tumor necrosis factor [TNF]α, and TNFβ) in serum samples were analyzed by multiplex assays (in Steps 1 and 2). Natural cytotoxicity (NK cell activity) of NK cells was assessed by measuring the ability of the patient’s peripheral blood mononuclear cells to lyse K562 cells in vitro before Cycle 1 in Step 2.

### Efficacy assessments

Tumor response to DS-8895a (best overall response, duration of best overall response, response rate, and disease control rate) was evaluated using the Response Evaluation Criteria in Solid Tumors, Version 1.1., using transverse section images of computerized tomography or magnetic resonance imaging scans. All baseline evaluations were performed using images taken within 21 days of registration. Tumor evaluation was performed every 6 weeks (± 1 week), or whenever investigators considered necessary.

### Statistical analysis

The Guidelines for the Clinical Evaluation of Anti-cancer Drugs [28] were used to determine the sample size for Step 1. For Step 2, the sample size of up to 20 patients was estimated to be sufficient to evaluate safety and efficacy of DS-8895a.

Summary statistics were calculated for all categorical and quantitative data. The Kaplan–Meier method was used to estimate the survival distribution function for time-to-event analyses. Progression-free survival (PFS) was defined as the time from the first dose of DS-8895a to progression, relapse, or death from any cause, whichever occurred first. Pre-planned analytical populations consisted of an efficacy analysis set (patients who received at least one dose of the study drug and had at least one tumor assessment), an MTD evaluable set (patients who received at least one dose of the study drug in Step 1), a PK and pharmacodynamics analysis set (patients who received at least one dose of the study drug and from whom appropriate samples were obtained), and a safety analysis set (patients who received at least one dose of the study drug). SAS® System Release 9.2 software (SAS Institute Inc., Cary, NC) was used to perform statistical analysis.

## Results

### Patients

In total, 37 patients (22 in Step 1 and 15 in Step 2) were enrolled from 7 November 2013 to 2 February 2017. The number of patients (total [Step 1, Step 2]) in each pre-planned analysis set was as follows: safety analysis set (37 [22, 15]), efficacy analysis set (36 [21, 15]), MTD evaluable analysis set (21, [21, 0]), PK analysis set (36 [21, 15]), and pharmacodynamics analysis set (37 [21, 15]). One patient in Step 1 (Level 6) was excluded from the efficacy, PK, and MTD analysis sets because of unavailable efficacy/PK data or unevaluable DLT due to an IRR, which prevented the patient from completing the initial DS-8895a administration/finishing the trial.

Patient characteristics are shown in Table 1 and were similar between patients in Steps 1 and 2. Overall, 76% of patients were male with an average age of 67 years. EPHA2 expression was positive (2+ and 3+) in approximately one-third of patients tested in Step 1. In Step 2, 72 patients were screened and 27 were positive for EPHA2 expression; 15 of these 27 patients met the eligibility criteria and were included in Step 2. All 37 enrolled patients received the study treatment and eventually discontinued the study. Most patients discontinued because of evident disease progression (20 in Step 1 and 13 in Step 2), and all others discontinued because of adverse events (Additional file 7).

**Table 1:** Patient characteristics

	Step 1	Step 2
	(*n* = 22)	(*n* = 15)
Sex, Male	15 (68)	13 (87)
Age, year (mean [range])	68 (56–78)	66 (49–76)
Weight, kg (mean [range])	55 (40–85)	55 (42–70)
Height, cm (mean [range])	162 (148–178)	165 (160–173)
Cancer type^a^		
Gastric	16 (73)	9 (60)
Esophageal	4 (18)	6 (40)
Colorectal	1 (5)	0 (0)
NSCLC	2 (9)	0 (0)
Thyroid	1 (5)	0 (0)
Primary tumor		
Present	11 (50)	8 (53)
Prior cancer therapy		
Chemotherapy	22 (100)	15 (100)
No. of regimens (mean [range])	4.4 (2–7)	4.5 (2–8)
Surgery	10 (46)	8 (53)
Radiation	3 (14)	4 (27)
EPHA2 expression^b^		
0	10 (46)	0 (0)
1+	2 (9)	0 (0)
2+	6 (27)	13 (87)
3+	1 (5)	2 (13)

N (%) unless otherwise stated

*Abbreviations*: NSCLC, non–small-cell lung cancer; EPHA2, erythropoietin-producing hepatocellular receptor A2

^a^Patients with more than one type of cancer were repeatedly counted

^b^Data were presented only for patients who have been examined for EPHA2 expression

### Safety

In Step 1, only one DLT was observed in one patient at Level 6 (20 mg/kg) as Grade 4 platelet count decreased. This was the only drug-related serious AE (SAE) and the only AE that required a dose delay in this study. MTD was not reached within the pre-planned doses. The dose for Step 2 was determined to be the highest dose from Step 1: 20 mg/kg.

AEs of any grade were observed in 21 (95.5%) and 15 (100%) patients in Steps 1 and 2, respectively (Table 2). The most common AEs were chills, decreased appetite, pyrexia, hypotension, nausea, anemia, hypoxia, constipation, dry skin, cancer pain, and vomiting. Drug-related AEs were observed in 14 patients (63.6%) in Step 1 and 10 patients (66.7%) in Step 2. The frequency of Grade 3 or 4 AEs was less than 10% in both Steps. IRRs related to DS-8895a occurred in 13 patients (59.1%) in Step 1 and 6 (40.0%) in Step 2. Among these patients, transient interruption of DS-8895a infusion was required in 10 patients. One patient in Level 3 (1.0 mg/kg) discontinued treatment because of a Grade 3 IRR, syncope. All other IRRs were Grade 1 or 2.

**Table 2:** Adverse events (AEs)

	Step 1	Step 2
	(n = 22)	(n = 15)
AEs, any grades	21 (95.5)	15 (100)
AEs, grade ≥ 3	5 (22.7)	8 (53.3)
Drug-related AEs, any grades	14 (63.6)	10 (66.7)
Drug-related AEs, grade ≥ 3	2 (9.1)	1 (6.7)
Syncope	1 (4.5)	0 (0.0)
Anemia	1 (4.5)	0 (0.0)
White blood cell count decreased	1 (4.5)	0 (0.0)
Lymphocyte count decreased	0 (0.0)	1 (6.7)
Neutrophil count decreased	1 (4.5)	0 (0.0)
Platelet count decreased^a^	1 (4.5)	0 (0.0)
Infusion-related reaction^b,c^	13 (59.1)	6 (40.0)
Chills	9 (40.9)	3 (20.0)
Grade 1	5 (22.7)	3 (20.0)
Grade 2	4 (18.2)	0 (0.0)
Pyrexia	5 (22.7)	4 (26.7)
Grade 1	4 (18.2)	4 (26.7)
Grade 2	1 (4.5)	0 (0.0)
Hypotension	5 (22.7)	2 (13.3)
Grade 1	2 (9.1)	2 (13.3)
Grade 2	3 (13.6)	0 (0.0)
Hypoxia	4 (18.2)	1 (6.7)
Grade 1	1 (4.5)	0 (0.0)
Grade 2	3 (13.6)	1 (6.7)
Nausea	4 (18.2)	0 (0.0)
Grade 1	3 (13.6)	0 (0.0)
Grade 2	1 (4.5)	0 (0.0)

N (%)

^a^Grade 4 platelet count decreased was judged as a dose-limiting toxicity

^b^Major reactions that occurred in four or more patients were presented

^c^No Grade 3 or 4 infusion-related adverse events occurred

In Steps 1 and 2, there were nine AEs in four patients that led to discontinuation of study treatment. Of these, AEs of hypoesthesia, hypotension, peripheral coldness, nausea, and vomiting occurred in a single patient on the day of the first dose and immediately resolved after cessation of DS-8895a infusion. These five events were determined as being related to DS-8895a while the remaining four AEs were symptoms associated with disease progression. Therefore, a single patient of the 37 patients enrolled in the study (2.7%) discontinued treatment due to DS-8895a-related toxicity. Eleven SAEs occurred in seven patients including three deaths due to disease progression, and all were unrelated to DS-8895a except for the Grade 4 platelet decreased previously mentioned.

No clinically relevant differences from baseline or consistent trends were observed for other laboratory parameters (blood chemistry, hematology, and urology), vital signs, and ECG.

### Pharmacokinetics

The mean maximum and trough serum concentrations of DS-8895a increased as the dose increased. PK parameters (C_max_ and AUC) increased with increasing dose of DS-8895a (Table 3) for both Cycles 1 and 2. No apparent trends were observed for T_max_. The mean T_1/2_ of DS-8895a was 10–14 days in patients treated with 1.0 mg/kg or higher.

**Table 3:** Pharmacokinetic parameters

	Step 1						Step 2
	0.1 mg/kg (N = 3)	0.3 mg/kg (*N* = 3)	1.0 mg/kg (N = 3)	3.0 mg/kg (N = 3)	10 mg/kg (N = 3)	20 mg/kg (*N* = 7)	20 mg/kg (*N* = 15)
Cycle 1, n	2	3	3	3	3	6	14
C_max_, μg/mL	1.70 ± 0.02	5.90 ± 1.18	21.3 ± 8.30	64.4 ± 15.2	169 ± 31.9	545 ± 170	369 ± 73.2
T_max_, h	3.88 (3.77, 4.00)	3.97 (1.05, 4.07)	3.96 (1.12, 7.00)	4.00 (3.97, 6.90)	6.73 (4.33, 6.97)	3.91 (2.25, 6.88)	3.43 (2.22, 7.00)
AUC_last_, μg·day/mL	0.86 ± 0.61	23.2 ± 6.03	124 ± 12.4	423 ± 29.7	943 ± 72.5	2570 ± 457	2340 ± 558
AUC_tau_, μg·day/mL	1.62 ± 1.03	24.4 ± 4.99	125 ± 12.1	423 ± 29.6	944 ± 73.1	2570 ± 460	2270 ± 534
AUC_inf_, μg·day/mL	NC^a^ ± NC^b^	33.1 ± 7.27	227 ± 15.8	671 ± 188	1530 ± 439	4520 ± 1910	4500 ± 1700
T_1/2_, day	NC^a^ ± NC^b^	4.94 ± 1.32	12.6 ± 4.43	9.69 ± 4.39	9.95 ± 3.62	11.6 ± 7.52	13.9 ± 6.60
Cycle 2, n	1	2	3	2	0	4	11
C_max_, μg/mL	1.60 ± NC^b^	9.54 ± 2.78	32.2 ± 2.12	86.8 ± 10.7	–	618 ± 151	489 ± 105
C_trough_, μg/mL	0.00 ± NC^b^	0.54 ± 0.76	10.5 ± 0.23	28.4 ± 0.50	–	188 ± 67.7	148 ± 54.6
T_max_, h	1.18 (1.18, 1.18)	5.48 (3.97, 7.00)	3.68 (1.13, 24.13)	5.44 (4.00, 6.88)	–	3.11 (2.13, 6.70)	4.00 (2.05, 6.88)
AUC_tau_, μg·day/mL	9.14 ± NC^b^	37.1 ± 5.93	259 ± 27.1	715 ± 13.8	–	4630 ± 1140	3500 ± 1140
T_1/2_, day	4.78 ± NC^b^	4.08 ± 0.91	13.3 ± 1.73	10.8 ± 0.13	–	12.4 ± 6.76	13.6 ± 8.14

Data are presented as mean ± standard deviation, or median (min, max), pharmacokinetic analysis set

*Abbreviations*: *C* concentration, *T* time, *AUC* area under the curve, *T*_*1/2*_ terminal elimination half-life, *NC* not calculated

^a^Date and time data of DS-8895a were not available so values were NC

^b^Two or fewer values were available so standard deviation could not be calculated

### Efficacy

**Fig. 1:**
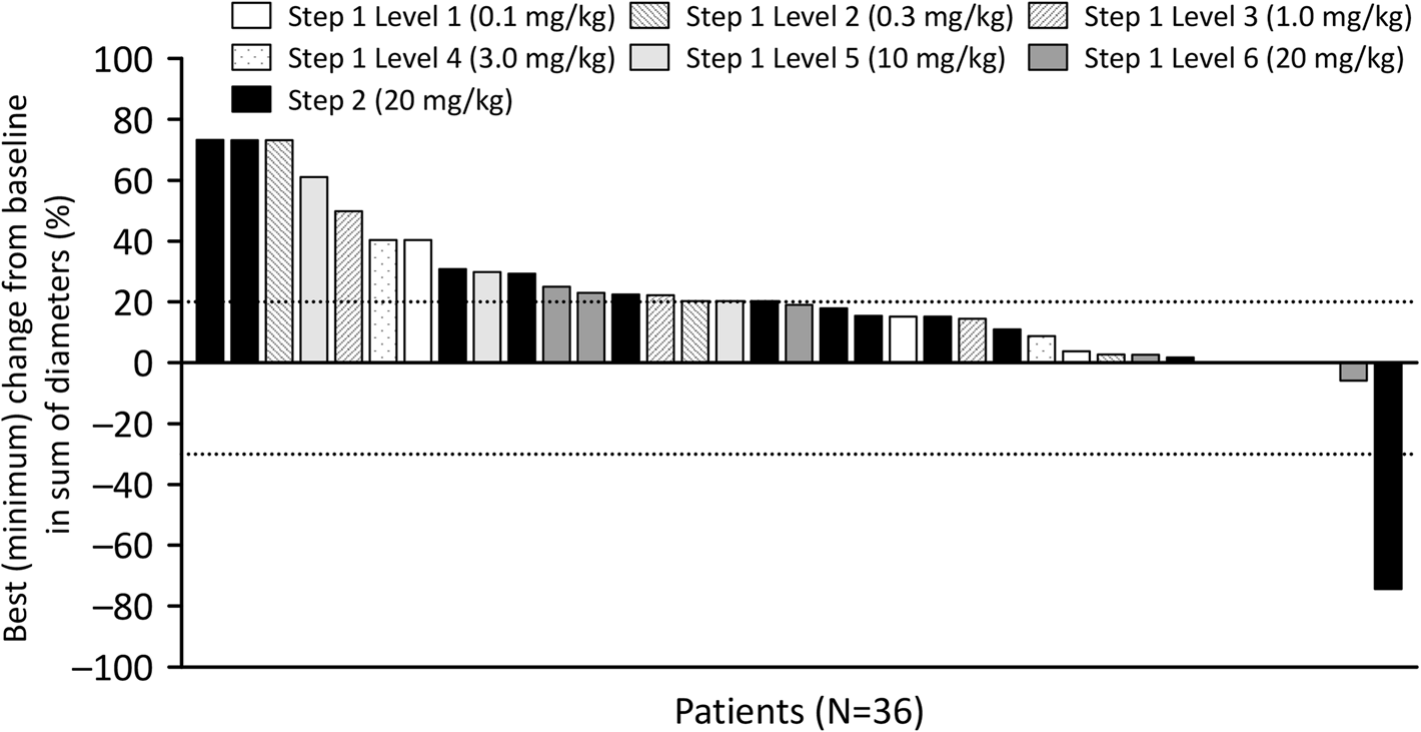
Best (minimum) percent change from baseline in sum of diameters (%) of target lesions. Baseline is defined as the last measurement prior to administration of the first dose of DS-8895a. Each vertical bar represents the best (minimum) percent change from baseline for an individual patient

One gastric cancer patient in Step 2 achieved partial response (PR) (6.7%). Stable disease (SD) was observed in seven patients (33.3%; 95% confidence interval [CI]: 14.6, 57.0) of Step 1 and six patients (40.0%; 95% CI: 16.3, 67.7) of Step 2 (Table 4). Tumor shrinkage demonstrated no clear correlation with DS-8895a dose (Fig. 1) or EPHA2 expression (Table 4).

**Table 4:** Efficacy results

	Step 1	Step 1 Unknown	Step 1 EPHA2^−^	Step 1 EPHA2^+^	Step 1 EPHA2^2+^	Step 1 EPHA2^3+^	Step 2	Step 2 EPHA2^−^	Step 2 EPHA2^+^	Step 2 EPHA2^2+^	Step 2 EPHA2^3+^
	(*N* = 21)	(*N* = 3)	(*N* = 9)	(N = 2)	(*N* = 6)	(N = 1)	(N = 15)	(*N* = 0)	(N = 0)	(*N* = 13)	(N = 2)
	*N* (%) [95% CI]	N (%) [95% CI]	N (%) [95% CI]	N (%) [95% CI]	N (%) [95% CI]	N (%) [95% CI]	N (%) [95% CI]	N (%) [95% CI]	N (%) [95% CI]	N (%) [95% CI]	N (%) [95% CI]
Complete response	0 (0.0) [0.0–16.1]	0 (0.0) [0.0–70.8]	0 (0.0) [0.0–33.6]	0 (0.0) [0.0–84.2]	0 (0.0) [0.0–45.9]	0 (0.0) [0.0–97.5]	0 (0.0) [0.0–21.8]	0 (0.0) [n.d.]	0 (0.0) [n.d.]	0 (0.0) [0.0–24.7]	0 (0.0) [0.0–84.2]
Partial response	0 (0.0) [0.0–16.1]	0 (0.0) [0.0–70.8]	0 (0.0) [0.0–33.6]	0 (0.0) [0.0–84.2]	0 (0.0) [0.0–45.9]	0 (0.0) [0.0–97.5]	1 (6.7) [0.2–31.9]	0 (0.0) [n.d.]	0 (0.0) [n.d.]	1 (7.7) [0.2–36.0]	0 (0.0) [0.0–84.2]
Stable disease	7^a^ (33.3) [14.6–57.0]	2 (66.7) [9.4–99.2]	3 (33.3) [7.5–70.1]	1 (50.0) [1.3–98.7]	1 (16.7) [0.4–64.1]	0 (0.0) [0.0–97.5]	6 (40.0) [16.3–67.7]	0 (0.0) [n.d.]	0 (0.0) [n.d.]	5 (38.5) [13.9–68.4]	1 (50.0) [1.3–98.7]
Progressive disease	14^a^ (66.7) [43.0–85.4]	1 (33.3) [0.8–90.6]	6 (66.7) [29.9–92.5]	1 (50.0) [1.3–98.7]	5 (83.3) [35.9–99.6]	1 (100.0) [2.5–100.0]	8 (53.3) [26.6–78.7]	0 (0.0) [n.d.]	0 (0.0) [n.d.]	7 (53.8) [25.1–80.8]	1 (50.0) [1.3–98.7]

*Abbreviations*: *EPHA2* erythropoietin-producing hepatocellular receptor A2, *CI* confidence interval, *n.d* not determined

^a^Includes patients with unknown EPHA2 status

The median (range) duration of study treatment was 4.1 (0.14–31) weeks for 22 patients in Step 1 and 5.1 (2.1–22) weeks for 15 patients in Step 2. Time to response of the PR patient was 5.1 weeks, and duration of response was 18.4 weeks. In the patients with PR and SD, the median (range) duration of disease control was 11.7 (5.0–34) weeks. The median (range) PFS for Steps 1 and 2 were 5.9 (4.0–34.3) and 6.0 (3.3–23.4) weeks, respectively.

### Pharmacodynamics

**Fig. 2:**
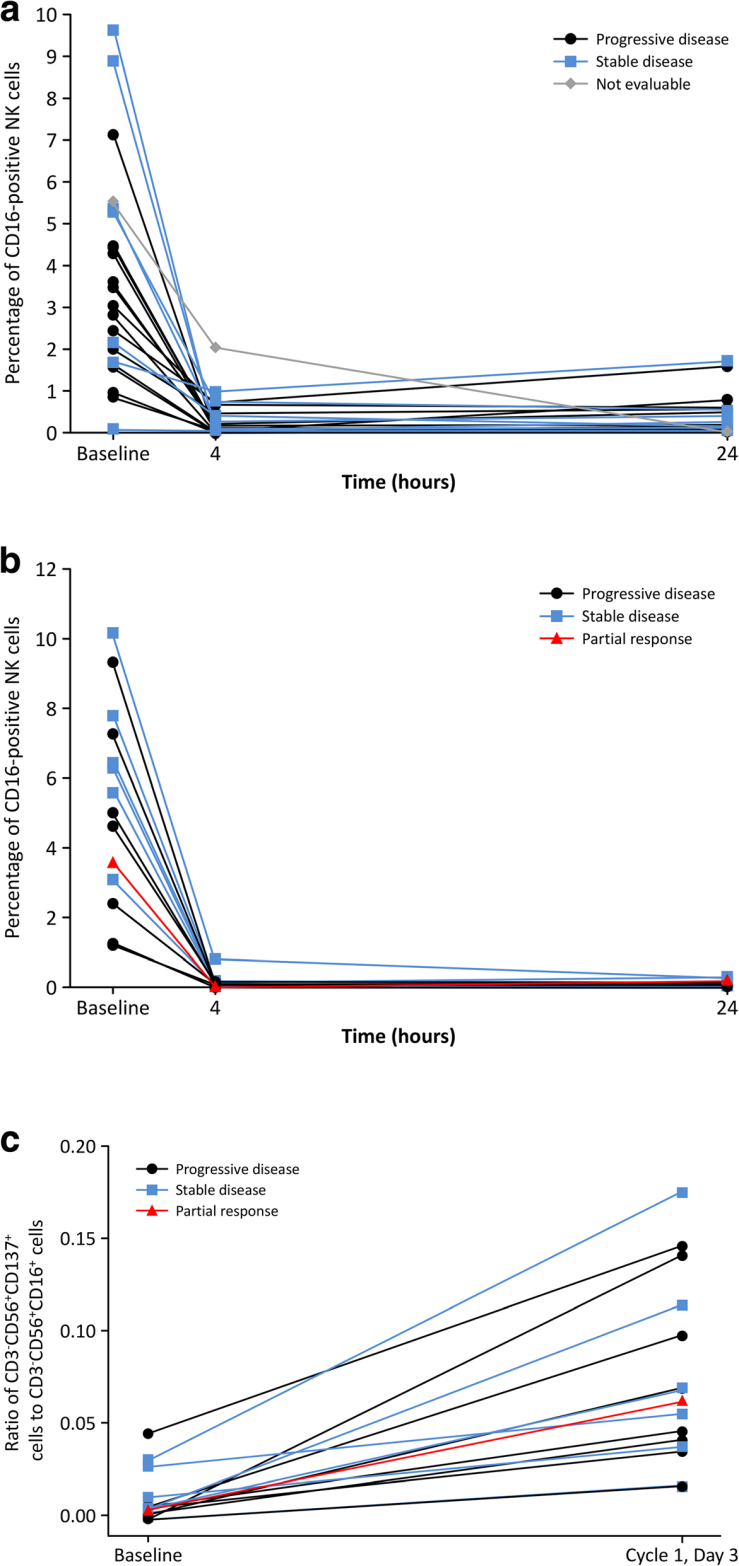
Changes in CD16-positive natural killer (NK) cells. Time-course of circulating CD16-positive NK cells (CD3^−^CD56^+^CD16^+^) after DS-8895a treatment in a) Cycle 1 of Step 1 and b) Cycle 1 of Step 2, and c) the ratio of CD3^−^CD56^+^CD137^+^ cells to CD3^−^CD56^+^CD16^+^ cells in Cycle 1 of Step 2

Serum levels of IFN-γ, IL-6, IL-7, IL-8, IL-10, MIP-1α, MIP-1β, MCP 1, and TNFα increased transiently at completion of DS-8895a administration and 4 h after the start of administration in Cycle 1. Elevated serum levels returned to baseline at 24 h. Soluble EPHA2 protein levels increased over time from baseline levels to day 8 of Cycle 2 in Step 2. Various levels of NK cell activity were observed in all patients before the first dose of DS-8895a in Step 2. Levels of circulating CD16-positive NK cells in the blood decreased 4 h after start of DS-8895a administration and remained low at 24 h in Cycle 1 of Step 1 (Fig. 2a) and Step 2 (Fig. 2b). The ratio of CD3^−^CD56^+^CD137^+^ cells to CD3^−^CD56^+^CD16^+^ cells increased on day 3 of Cycle 1 in Step 2, compared with baseline (Fig. 2c). There were no apparent relationships between the levels or changes of these biomarkers and tumor responses.

Mismatch of *HLA/KIR* polymorphism was detected in 20 patients (2 matched) in Step 1 and 14 patients (1 matched) in Step 2. All three patients with matched *HLA/KIR* had progressive disease. No association with tumor response could be determined because of the high incidence of *HLA/KIR* mismatch.

EPHA2, E-cadherin, HER2, and EGFR expression in retained tumor tissues; and EPHA2, CD16, NKp46/NCRI, CD3, CD68, PD-L1, E-cadherin, EGFR, and HER2 expression in paired biopsies obtained before Cycle 1 and 2 also demonstrated no apparent relationship between these expression levels or changes in expression levels and tumor responses. Pre- and post-treatment tumor tissues from the patient who achieved PR showed high expression of PD-L1 and E-cadherin at baseline, which decreased after DS-8895a administration. Also, in this patient, the number of infiltrated CD3-positive cells increased after DS-8895a treatment.

## Discussion

Here we report the results of the first-in-human study of DS-8895a, an afucosylated, humanized anti-EPHA2 antibody. DS-8895a was safe and well tolerated up to 20 mg/kg in patients with advanced solid tumors (Step 1) and EPHA2-positive gastric or esophageal cancer (Step 2). Although one DLT event was observed at 20 mg/kg in Step 1, the MTD was not reached. The safety of a 20 mg/kg dose level was further confirmed in Step 2. IRRs were reported in about half of the patients but were generally manageable. One patient with EPHA2-positive gastric cancer achieved PR and 13 patients showed SD as best response. Serum DS-8895a concentrations and exposure increased in a dose-dependent manner, as expected. The mean T_1/2_ of DS-8895a was 10–14 days in patients treated with 1.0 mg/kg or higher and a stable concentration level was reached after long-term treatment, suggesting that administration every 2 weeks is a reasonable treatment schedule.

In this study, a decrease in CD16-positive NK cells and a transient increase in serum inflammatory cytokines were observed after DS-8895a administration, both of which indicate DS-8895a ADCC activity. Reduction of CD16-positive NK cells was maintained for 24 h, which was consistent with reports of other ADCC-enhanced antibodies [29, 30], suggesting a decrease in CD16-positive NK cells by ADCC. The increase in cytokines might also contribute to the occurrence of IRRs, which has been reported for other types of afucosylated monoclonal antibodies [31, 32].

We also observed that the ratio of CD3^−^CD56^+^CD137^+^ cells to CD3^−^CD56^+^CD16^+^ cells increased from baseline to Day 3 of Cycle 1. It has been reported that induction of ADCC activity upregulates CD137 expression on NK cells [33, 34], and increased CD137 expression on circulating NK cells has also been identified in patients treated with cetuximab, an anti-EGFR antibody with ADCC activity [35]. However, no apparent relationship was noted between this ratio and the best overall response in this study, given that we had only one patient who achieved PR. This suggests that enhanced ADCC activity by our afucosylated antibody was not sufficient to induce strong tumor shrinkage in solid tumors. The addition of an agonistic anti-CD137 monoclonal antibody with other antibodies, such as cetuximab or anti-CD20 antibody, has shown that activation of CD137 on NK cells enhanced their antitumor activity [34, 35]. A previous study suggested that the combination of *HLA* and *KIR* gene polymorphisms may affect ADCC activity [36]. However, as *HLA/KIR* mismatch was present in most patients in both Step 1 and Step 2, we were unable to assess a relationship between *HLA/KIR* mismatch and ADCC activity in our study.

Additional investigations of pharmacodynamic biomarkers conducted in Step 2 — including NK cell activity before the first study treatment and tumor expression levels of EPHA2, CD16, NKp46/NCRI, CD3, CD68, PD-L1, E-cadherin, EGFR, and HER2 — yielded no apparent trends of correlation between baseline level or on-treatment changes of these biomarkers and best overall response or disease control ratio. In Step 2, patients with EPHA2-positive gastric or esophageal cancer were enrolled. However, only one patient achieved a response, indicating that patient enrichment or drug activity may not have been sufficient. The patient who achieved PR had higher PD-L1 expression at baseline compared with the other patients and showed an increase in CD3-positive cells in Cycle 2 and decreased PD-L1 expression compared with baseline levels. Activated NK cells can stimulate the activity of other immune processes through their release of cytokines (such as IFNγ), providing a link to initiate subsequent immune responses to attack target tumors, which may have resulted in a tumor response in this patient. Preclinical studies using combination treatment of DS-8895a with other agents such as immune check point inhibitors are warranted.

As the first-in-human study of DS-8895a, this study has provided initial insights into the safety and potential activity of DS-8895a in patients and their response to the drug, providing a valuable knowledge base for future studies of afucosylated, humanized antibodies for the treatment of solid tumors. While clinical recommendations cannot be made based on this early-stage study, our phase I clinical study results warrant further studies involving a greater number of patients to determine the significance of our reported observations in relation to the treatment of EPHA2-positive solid tumors. Exploratory pharmacodynamic analysis suggested that immunological change was induced by our afucosylated monoclonal antibody with enhanced ADCC activity, which warrants further investigations to assess the efficacy of DS-8895a with different combinations of immune checkpoint inhibitors.

### Limitations

One limitation of this study was that only Japanese patients were involved; therefore, the generalizability of the findings to other ethnic populations may be limited. Additionally, the PFS results from this single-arm study (without comparators) are considered exploratory.

## Conclusions

This study showed that 20 mg/kg DS-8895a administered by infusion every 2 weeks was generally safe and well tolerated in patients with advanced solid tumors and that serum concentrations of DS-8895a increased in a dose-dependent manner. While no associations between biomarker changes and best overall response were observed, it will be of particular interest to further investigate changes in the ratio of CD137^+^ NK cells to NK cells after DS-8895a treatment to understand if an increased ratio may correlate with a positive treatment response or if the addition of agonistic anti-CD137 monoclonal antibodies may enhance the treatment response in patients with increased CD137 expression on NK cells.

## Additional files

Additional file 1: Schematic of the cytotoxic effect of DS-8895a. Abbreviations: EPHA2, erythropoietin-producing hepatocellular receptor A2; FcγRIIIa, Fragment crystallizable gamma receptor IIIa; NK, natural killer. (TIF 785 kb)

Additional file 2: Selection criteria: sufficient organ function (DOCX 13 kb)

Additional file 3: Description of treatment-free period (DOCX 14 kb)

Additional file 4: Overall study design. Abbreviations: PK, pharmacokinetics; Q2W, every 2 weeks. (TIF 542 kb)

Additional file 5: Definition of dose-limiting toxicities (DOCX 15 kb)

Additional file 6: Schedule of blood/serum collection (DOCX 16 kb)

Additional file 7: Disposition of patients. ^†^One patient was excluded from the efficacy analysis set because no efficacy data were available. ^‡^One patient was excluded from the PK analysis set because no PK data were available. Abbreviation: PK, pharmacokinetics. (TIF 224 kb)

## Data Availability

The datasets supporting the conclusions of this article are included within the article and its additional files.

## Abbreviations

ADCC: Antibody-dependent cellular cytotoxicity
AE: Adverse event
AUC: Area under the concentration-time curve
CI: Confidence interval
C_max_: Maximum serum concentration
DLT: Dose-limiting toxicity
ECG: Electrocardiogram
EGFR: Epidermal growth factor receptor
EPHA2: Erythropoietin-producing hepatocellular receptor A2
EPHRIN-A1: EPH-related receptor tyrosine kinase ligand A1
FcγRIIIa: Fragment crystallizable gamma receptor IIIa
HER2: Human epidermal growth factor receptor 2
HLA: Human leukocyte antigen
IFN: Interferon
IL: Interleukin
IRR: Infusion-related reaction
KIR: Killer cell immunoglobin-like receptor
MCP: Monocyte chemotactic protein
MIP: Macrophage inflammatory protein
MTD: Maximum tolerated dose
NK: Natural killer
PD-L1: Programmed death ligand-1
PFS: Progression-free survival
PK: Pharmacokinetics
PR: Partial response
SAE: Serious adverse event
SD: Stable disease
T_1/2_: Terminal elimination half-life
T_max_: Time to reach maximum serum concentration
TNF: Tumor necrosis factor
