# Supplementary figures and images for: Safety, tolerability, pharmacokinetics, and pharmacodynamics of the afucosylated, humanized anti-EPHA2 antibody DS-8895a: a first-in-human phase I dose escalation and dose expansion study in patients with advanced solid tumors
Source: J Immunother Cancer. 2019 Aug 14;7:219. doi: 10.1186/s40425-019-0679-9 (PMC6694490; doi:10.1186/s40425-019-0679-9)

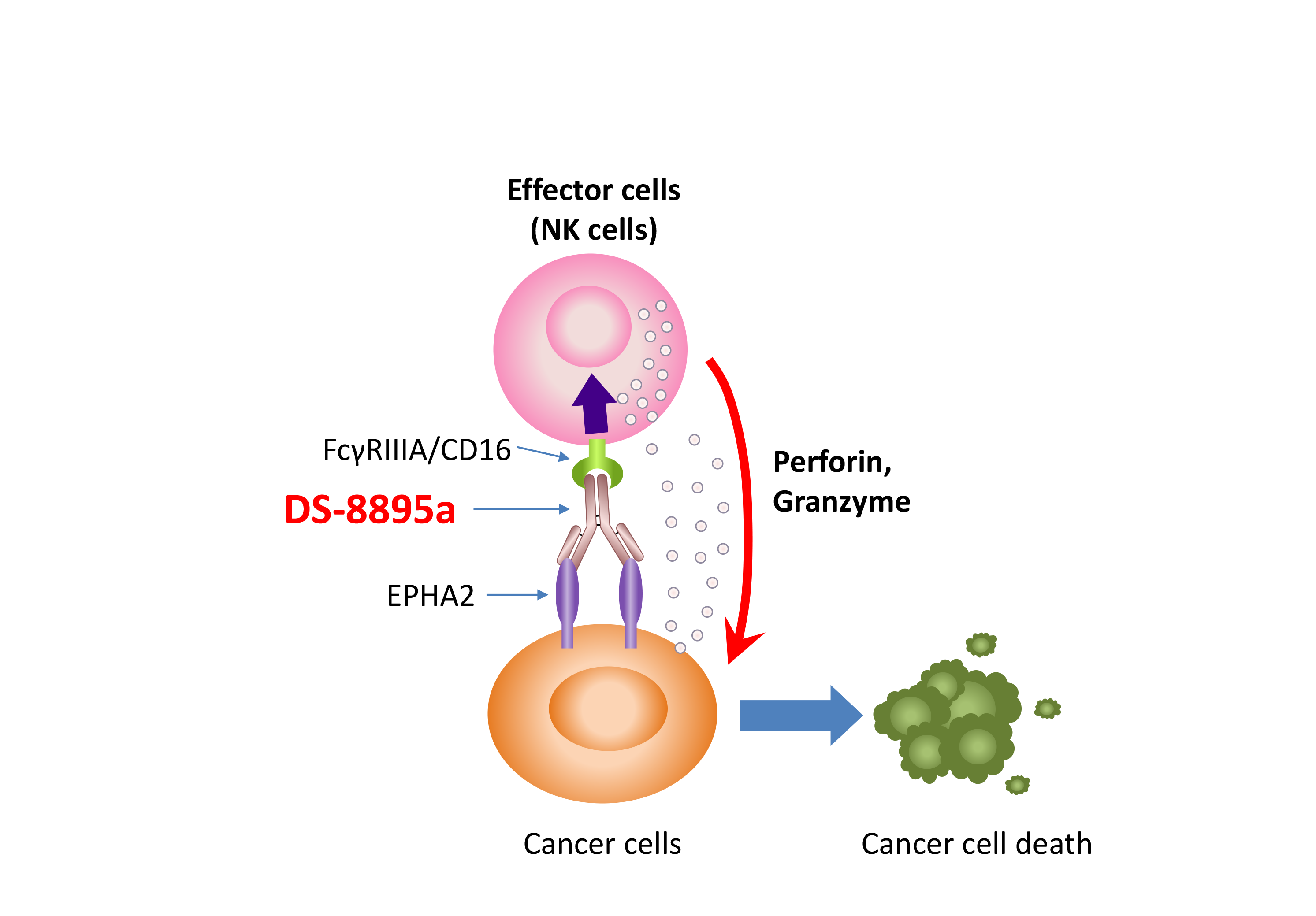

Supplement: Supplementary file 1 — Schematic of the cytotoxic effect of DS-8895a. Abbreviations: EPHA2, erythropoietin-producing hepatocellular receptor A2; FcγRIIIa, Fragment crystallizable gamma receptor IIIa; NK, natural killer. (TIF 785 kb) [file 40425_2019_679_MOESM1_ESM.tif]

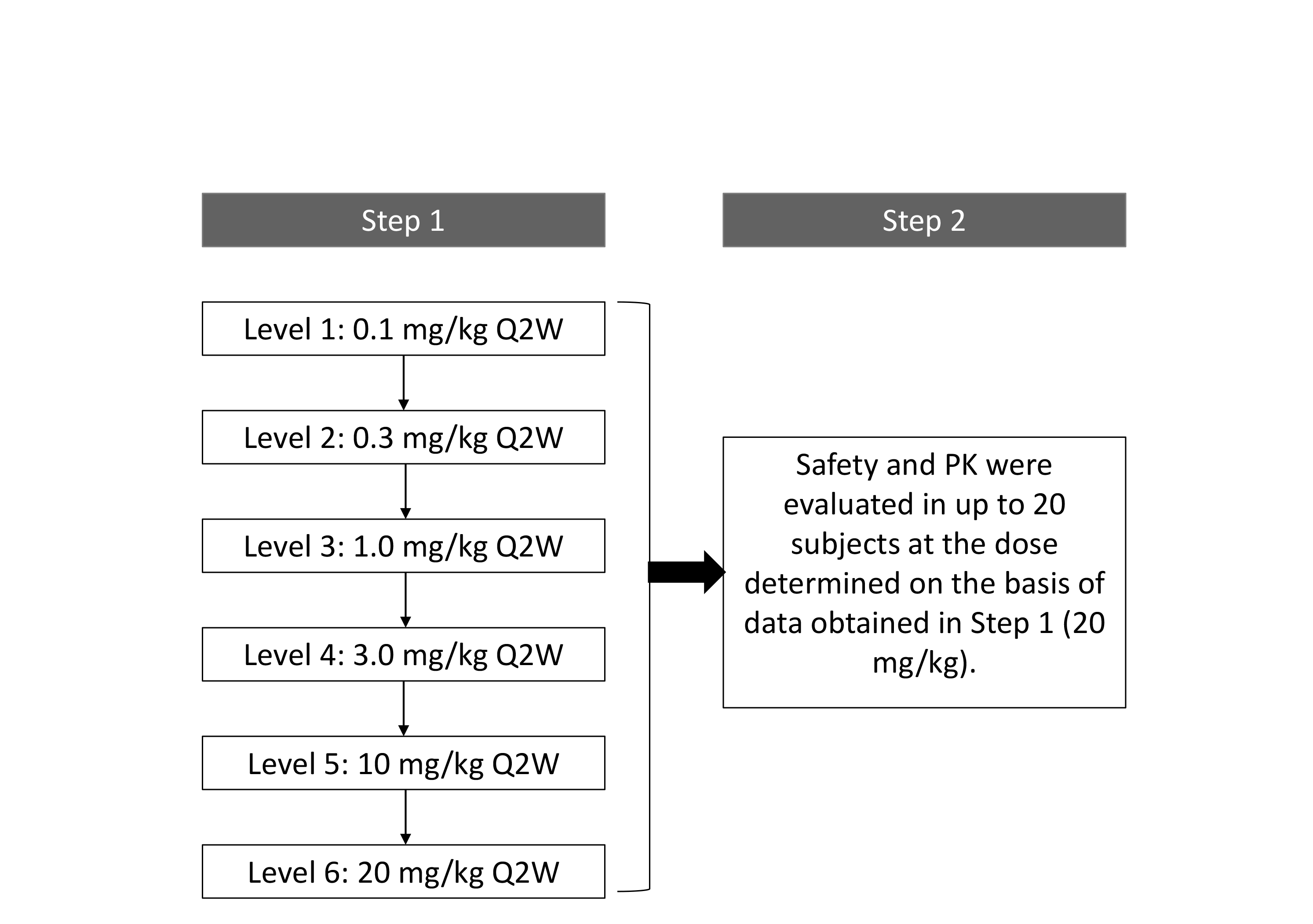

Supplement: Supplementary file 4 — Overall study design. Abbreviations: PK, pharmacokinetics; Q2W, every 2 weeks. (TIF 542 kb) [file 40425_2019_679_MOESM4_ESM.tif]

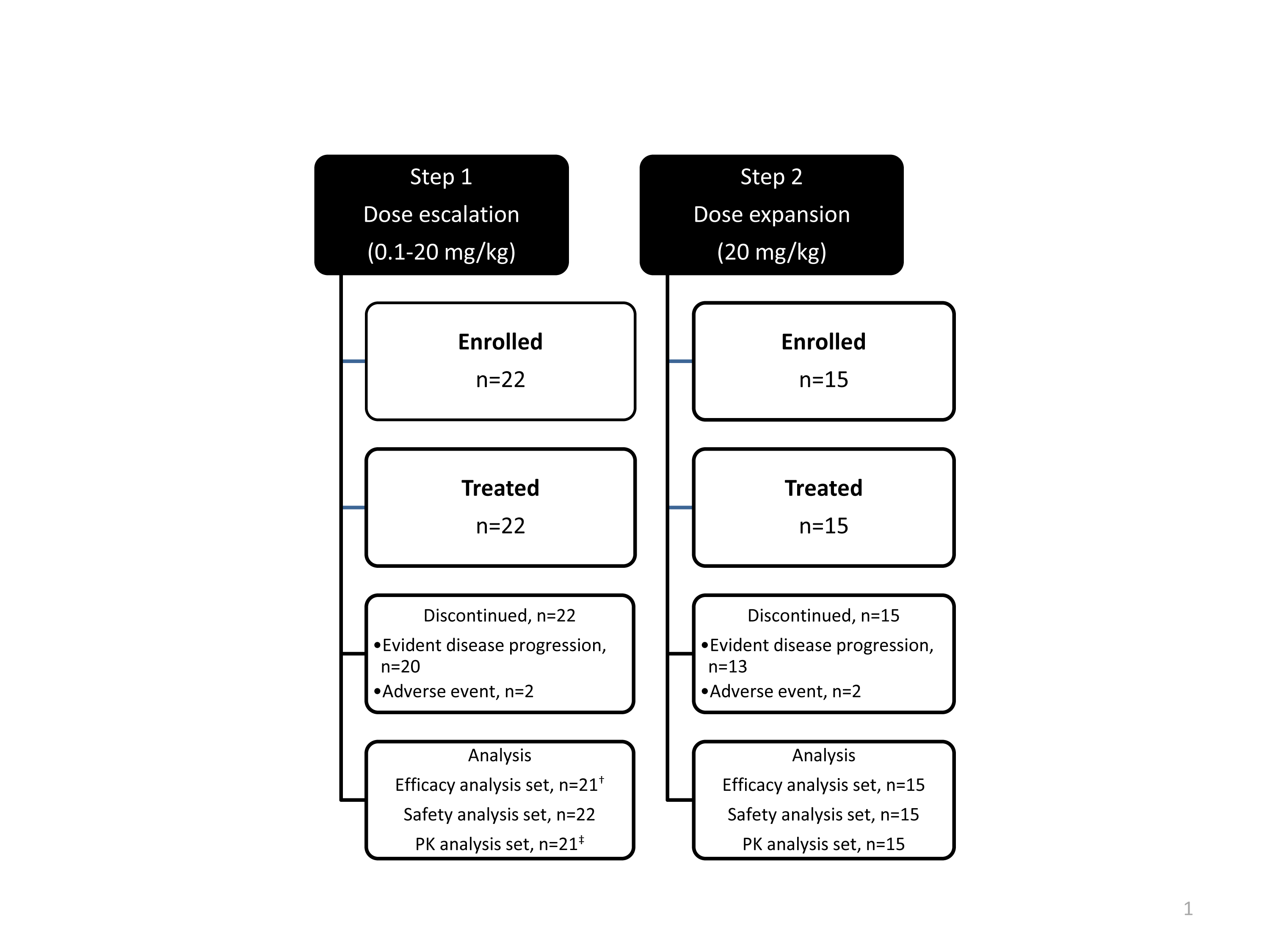

Supplement: Supplementary file 7 — Disposition of patients. †One patient was excluded from the efficacy analysis set because no efficacy data were available. ‡One patient was excluded from the PK analysis set because no PK data were available. Abbreviation: PK, pharmacokinetics. (TIF 224 kb) [file 40425_2019_679_MOESM7_ESM.tif]
