# Supplementary material for: Safety, tolerability, pharmacokinetics, and pharmacodynamics of the afucosylated, humanized anti-EPHA2 antibody DS-8895a: a first-in-human phase I dose escalation and dose expansion study in patients with advanced solid tumors
Source: J Immunother Cancer. 2019 Aug 14;7:219. doi: 10.1186/s40425-019-0679-9 (PMC6694490; doi:10.1186/s40425-019-0679-9)
Supplement: Supplementary file 2 — Selection criteria: sufficient organ function (DOCX 13 kb) [file 40425_2019_679_MOESM2_ESM.docx]

**Additional file 2.** Selection criteria: sufficient organ function

| **Parameter** | **Acceptance Criteria** |
| --- | --- |
| Neutrophil count  Platelet count  Hemoglobin  AST  ALT  Total bilirubin  Serum creatinine  PT-INR | ≥ 1500/μL  ≥ 100 000/μL  ≥ 8.0 g/dL  ≤ 3 × ULN  (if liver metastases are present, ≤ 5 × ULN)  ≤ 3 × ULN  (if liver metastases are present, ≤ 5 × ULN)  ≤ 1.5 × ULN  ≤ 1.5 × ULN  ≤ 1.5 × ULN |

Abbreviations: AST, aspartate aminotransferase; ALT, alanine aminotransferase; ULN, upper limit of normal; PT-INR, prothrombin time-international normalized ratio
