# Supplementary material for: Safety, tolerability, pharmacokinetics, and pharmacodynamics of the afucosylated, humanized anti-EPHA2 antibody DS-8895a: a first-in-human phase I dose escalation and dose expansion study in patients with advanced solid tumors
Source: J Immunother Cancer. 2019 Aug 14;7:219. doi: 10.1186/s40425-019-0679-9 (PMC6694490; doi:10.1186/s40425-019-0679-9)
Supplement: Supplementary file 3 — Description of treatment-free period (DOCX 14 kb) [file 40425_2019_679_MOESM3_ESM.docx]

**Additional file 3.** Description of treatment-free period

The treatment-free period from the final dose/treatment of any previous therapy to the date of registration was as follows:

- Chemotherapy (including antibody drugs), 3 weeks
  - Nitrosourea or mitomycin C, 6 weeks
- Hormonal therapy, 3 weeks
- Radiation therapy, 3 weeks
  - Irradiation for bone metastasis (excluding pelvic irradiation) for pain relief and brain metastasis, 2 weeks
- Surgical therapy, 3 weeks
  - 2 weeks for less-invasive surgical procedures such as ostomy
