# Supplementary material for: Safety, tolerability, pharmacokinetics, and pharmacodynamics of the afucosylated, humanized anti-EPHA2 antibody DS-8895a: a first-in-human phase I dose escalation and dose expansion study in patients with advanced solid tumors
Source: J Immunother Cancer. 2019 Aug 14;7:219. doi: 10.1186/s40425-019-0679-9 (PMC6694490; doi:10.1186/s40425-019-0679-9)
Supplement: Supplementary file 5 — Definition of dose-limiting toxicities (DOCX 15 kb) [file 40425_2019_679_MOESM5_ESM.docx]

**Additional file 5.** Definition of dose-limiting toxicities

The dose-limiting toxicity (DLT) evaluation period was the 28 days following the first dose of DS-8895a (Step 1 only) and patients were hospitalized at least during the DLT evaluation period. DLT was defined as the following toxicities assessed as related to DS-8895a:

1. Hematological toxicity
   1. Grade 4 neutrophil count decreased persisting for 7 days or longer
   2. Grade 3 or more severe febrile neutropenia
   3. Grade 4 anemia
   4. Grade 4 platelet count decreased, or Grade 3 platelet count decreased requiring blood transfusion
2. Non-hematological toxicity
   1. Grade 3 or more severe fatigue persisting for 3 days or longer
   2. Grade 3 or more severe nausea, vomiting, diarrhea, or anorexia persisting for 3 days or longer despite the maximum supportive care
   3. Abnormal hepatic function that met any of the following:
      1. Grade 4 or more severe increase in aspartate aminotransferase (AST) and/or alanine aminotransferase (ALT)
      2. Grade 3 or more severe increase in AST and/or ALT and Grade 2 or more severe increase in blood bilirubin
      3. Grade 3 or more severe increase in AST and/or ALT persisting for 4 days or longer in subjects without liver metastasis
      4. Grade 3 or more severe increase in AST and/or ALT persisting for 4 days or longer in subjects with liver metastasis who had Grade 1 or less severe increase in AST and/or ALT before the start of the study treatment
      5. Increase in AST and/or ALT more than eight times the upper limit of normal persisting for 4 days or longer in subjects with liver metastasis who had Grade 2 increase in AST and/or ALT before the start of the study treatment
   4. Grade 3 or more severe non-hematological toxicities, excluding (a) through (c) shown above, and the following:
      1. Electrolytes abnormal, resolving within 3 days after onset
      2. Grade 3 or more severe increase in alkaline phosphatase and uric acid that were asymptomatic and resolved within 3 days after onset

Toxicity grading was in accordance with National Cancer Institute Common Terminology Criteria for Adverse Events (NCI-CTCAE) Version 4.0.

If any of the above toxicities was reported during the DLT evaluation period, the decision to regard the toxicity as a DLT was determined based on consultation between the investigator and sponsor. In addition, regarding other toxicities that would hinder the conduct of the scheduled study treatment, the decision to label them as DLTs was determined based on consultation between the investigator and sponsor. Infusion-related reaction, regardless of its grade, was excluded from evaluation of DLT in principle; severity of an infusion-related reaction depends upon the level of responsiveness of individual subjects and has no correlation with the dose of DS-8895a. However, if the sponsor or investigator assessed infusion-related reactions as requiring further assessment on the basis of the frequency of its occurrence in individual patients, proportion of affected patients, severity, etc., the decision of whether to consider the infusion-related reaction as a DLT was determined based on consultation between the investigator and sponsor. The sponsor was to consult with the medical expert as required in determining DLTs.
