# Supplementary material for: Safety, tolerability, pharmacokinetics, and pharmacodynamics of the afucosylated, humanized anti-EPHA2 antibody DS-8895a: a first-in-human phase I dose escalation and dose expansion study in patients with advanced solid tumors
Source: J Immunother Cancer. 2019 Aug 14;7:219. doi: 10.1186/s40425-019-0679-9 (PMC6694490; doi:10.1186/s40425-019-0679-9)
Supplement: Supplementary file 6 — Schedule of blood/serum collection (DOCX 16 kb) [file 40425_2019_679_MOESM6_ESM.docx]

**Additional file 6.** Schedule of blood/serum collection

| **Sample purpose** | **Administration** | **Day** | **Sampling time-point (acceptable range)** |
| --- | --- | --- | --- |
| Blood: circulating CD16-positive NK cells | Cycles 1 and 2 | 1 | Before administration (day of administration)  4 h after start of administration (± 20 min) |
|  |  | 2 | 24 h after start of administration (± 2 h) |
| Blood: *HLA/KIR* mismatch,  NK activity (Step 2 only) | - | - | Before the first dose of DS-8895a |
| Blood: blood cell markers, marker for blood cell activation (Step 2 only) | - | - | Before the first dose of DS-8895a |
|  | Cycle 1 | 3 | 48 h after start of administration (± 2 h) |
| Serum: Cytokines | Cycles 1 and 2 | 1 | Before administration (day of administration)  At completion (± 15 min)  4 h after start of administration (± 20 min)  7 h after start of administration (± 20 min) |
|  |  | 2 | 24 h after start of administration (± 2 h) |
| Serum: Soluble EPHA2 | Cycle 1 | 1 | Before administration (day of administration) |
| (Step 2 only) |  | 4 | 72 h after start of administration (± 2 h) |
|  |  | 8 | (± 1 day) |
|  |  | 15 | Before administration (day of administration) |
|  | Cycle 2 | 8 | (± 1 day) |

Abbreviations: NK cells, natural killer cells; HLA, human leukocyte antigen; KIR, killer cell immunoglobulin-like receptor; EPHA2; erythropoietin-producing hepatocellular receptor A2
